# Serum neurofilament light chain associates with symptom burden in Lyme neuroborreliosis patients: a longitudinal cohort study from Norway

**DOI:** 10.1007/s00415-024-12237-z

**Published:** 2024-02-26

**Authors:** Ingerid Skarstein, Elling Ulvestad, Anne Marit Solheim, Christian Vedeler, Unn Ljøstad, Åse Mygland, Randi Eikeland, Harald Reiso, Åslaug Rudjord Lorentzen, Steffan Daniel Bos

**Affiliations:** 1 Department of Microbiology, Haukeland University Hospital, Post Box 1400, 5021 Bergen, Norway; 2 Department of Clinical Science, University of Bergen, Bergen, Norway; 3 Department of Neurology, Sørlandet Hospital Trust, Kristiansand, Norway; 4 Department of Clinical Medicine, University of Bergen, Bergen, Norway; 5 Department of Neurology, Haukeland University Hospital, Bergen, Norway; 6 Section of Habilitation, Sørlandet Hospital Trust, Kristiansand, Norway; 7 Norwegian National Advisory Unit on Tick-Borne Diseases, Sørlandet Hospital Trust, Kristiansand, Norway; 8 Faculty of Health and Sport Sciences, University of Agder, Grimstad, Norway; 9 Department of Neurology, Oslo University Hospital, Oslo, Norway; 10 Cancer Registry of Norway, The Norwegian Institute of Public Health, Oslo, Norway

**Keywords:** Lyme neuroborreliosis, Tick-borne diseases, Neurofilament light chain, Central nervous system infections

## Abstract

**Objectives:**

Serum neurofilament light chain (sNfL), an indicator of neuronal damage, is increasingly recognized as a potential biomarker for disease activity in neurodegenerative disorders. In this study, we wanted to investigate sNfL as a prognostic marker in a large, well-defined population of 90 patients with Lyme neuroborreliosis (LNB). In addition, we sought to explore associations between symptoms and sNfL levels during the acute phase of LNB.

**Materials and methods:**

Patients diagnosed with definite or possible LNB were recruited from a double-blinded, placebo-controlled, multi-center trial, in which the participants were randomly assigned to 2 or 6 weeks of oral doxycycline treatment. The sNfL levels were measured using a single molecule array assay at both diagnosis and 6-month follow-up, and analysed against clinical parameters, variations in symptom burden and long-term complaints as assessed by a composite clinical score.

**Results:**

At the time of diagnosis, approximately 60% of the patients had elevated sNfL levels adjusted for age. Notably, mean sNfL levels were significantly higher at diagnosis (52 pg/ml) compared to 6 months after treatment (12 pg/ml, *p* < 0.001), when sNfL levels had normalized in the majority of patients. Patients with objective signs of spinal radiculitis had significantly higher baseline sNfL levels compared to patients without spinal radiculitis (*p* = 0.033).

**Conclusion:**

Our findings suggest that sNfL can serve as a biomarker for peripheral nerve tissue involvement in the acute phase of LNB. As found in an earlier study, we confirm normalization of sNfL levels in blood after treatment. We found no prognostic value of acute-phase sNfL levels on patient outcome.

## Introduction

Lyme neuroborreliosis (LNB), the most common form of disseminated Lyme borreliosis in Europe, is caused by spirochetes within the *Borrelia burgdorferi* sensu lato complex (Bb) [1–3]. Although Bb infection can affect both the peripheral and central nervous systems (PNS and CNS, respectively), the most common symptoms originate from the PNS in the form of lymphocytic meningitis, radiculitis, and unilateral peripheral facial palsy. Symptoms from the CNS such as encephalitis and myelitis are rare [3–6].

Much effort has been put into the development of molecular methods for detection of Bb in various tissues and during different disease manifestations. The polymerase chain reaction (PCR) has been found useful for detection of Bb in skin biopsies and synovial fluid, where high numbers of Bb can be present. In contrast, the sensitivity of PCR in serum and cerebrospinal fluid (CSF) is much lower, due to small numbers of Bb [7–9]. Diagnosis of LNB is, therefore, to a large degree dependent on the combination of clinical findings, CSF pleocytosis and intrathecal Bb antibody production.

Although previous studies have shown that most patients recover after appropriate treatment for LNB, a proportion of patients report long-term complaints [10–14]. The causal nature of such complaints is obscure, much because specific and objective tests for disease activity and prognosis are lacking. The few existing objective tests that indicate disease activity, including normalization of cell counts in CSF after treatment, do not provide clinicians with adequate tools for monitoring treatment and disease activity. There is, therefore, a demand for other disease-related biomarkers.

Neurofilaments are a group of proteins exclusively located in neurons, both in the PNS and CNS. They consist of four subunits: neurofilament heavy chain, medium chain, light chain, and one subunit dependent on location; alpha-internexin in the CNS and peripherin in the PNS [15, 16]. Neurofilament heavy and light chains are released into CSF and serum following axonal degeneration or injury. The levels of neurofilament light chain (NfL) are highly correlated between serum and CSF, making serum NfL (sNfL) an increasingly utilized biomarker in neurodegenerative diseases [17–20]. To the best of our knowledge, there is only one prior study of sNfL in LNB [21]. This study demonstrated a reduction in sNfL levels following antibiotic treatment for LNB, although the level of sNfL in the acute phase did not predict patient outcomes.

The present study was conducted to further investigate the sNfL as prognostic marker in a large, well-defined population of patients with LNB, and to look for associations between symptoms and sNfL levels in the acute phase of LNB.

## Materials and methods

### Study design

Patients above 18 years of age with neurological symptoms and/or findings suggestive of LNB were recruited to a randomized, double-blinded, placebo-controlled, multi-center, non-inferiority designed study (“Lyme borreliosis; a scientific approach to reduce diagnostic and therapeutic uncertainties”—*BorrSci*), with the aim to investigate the efficacy of 2 versus 6 weeks of treatment with 200 mg oral doxycycline once daily. Concentrations of leukocytes, immunoglobulin G (IgG), and antibodies against Bb were measured as part of the diagnostic procedure. Definite LNB was defined by presence of both CSF pleocytosis and intrathecally produced borrelia-specific antibodies, whereas presence of only CSF pleocytosis or positive Bb antibody index in a patient with clinical symptoms typical of LNB was defined as possible LNB [22]. The 121 included patients were recruited from 8 hospitals in Southern Norway between 2015 and 2020, and they were followed up for 12 months after recruitment [12, 23]. Serum samples were drawn at the time of inclusion and at 6 months and 12 months follow-up. For the present investigations, we included 90 patients from which we had serum from both inclusion and 6-month follow-up available at the time the analyses were done, 45 patients from each treatment arm. The 180 serum samples had been stored at -80 ͦC upon processing and had not been freeze-thawed after initial biobanking.

### Clinical characterization

Patient symptoms and complaints related to LNB were registered at inclusion, 10 weeks, 6 months and 12 months. For each patient, we assessed a composite clinical score (CCS) consisting of 10 physician scored questions of subjective complaints and 22 objective neurological findings, 12 from the PNS and 10 from the CNS [12]. A radicular sensory finding or radicular paresis was defined as a sensibility loss or a paresis matching a peripheral nerve or plexus. The CCS is described in detail by Solheim et al. [23]. Each item was scored 0–2 points, where 0 was defined as no symptoms, 1 as mild symptoms, and 2 as severe symptoms. Mild symptoms were defined as “without influence on daily life”, whereas severe symptoms were defined as “with influence on daily life”. Ergo, the CCS sum score ranged from 0 to 64 points [12]. Clinical data were registered in an electronic case report form (Viedoc technologies, Uppsala, Sweden).

### Measurement of neurofilament light chain in serum

The sNfL levels were measured using the bead-based immunoassay Simoa NF-light V2 Advantage Kit from Quanterix Corporation (Billerica, Massachusetts). Briefly explained, patient serum was incubated with paramagnetic beads covered with sNfL-specific antibodies. After washing and addition of detection conjugate, the beads for each sample were transferred to the Simoa disc containing 200,000 wells—each with room for one bead—and then analysed by the Simoa HD-X AnalyzerTM instrument. The lower limit of quantification in the lot used for this study was 2.56 pg/ml. All samples were analysed in duplicate in the same run, and mean concentrations calculated for further analysis. Intra-assay coefficients of variation were below 20%.

### Statistical analysis

All statistical analyses were performed using SPSS (IBM SPSS version 29).

Categorical variables are presented by counts and percentages, and continuous variables by median and interquartile range (IQR) or means and confidence intervals (CI).

Where suitable, sNfL levels were log_10_ transformed to obtain an approximately normal distribution. For comparison of transformed sNfL levels, we performed the independent samples *t* test, and where relevant a paired samples *t* test. For comparison of non-quantitative or not normally distributed variables, the Mann–Whitney *U* test was used. A two-sided *p* value (*p*) < 0.05 was considered as statistically significant regardless of test. For exploration of the correlations between sNfL levels and dichotomized outcomes, we used logistic regression, with the dichotomized outcome as the dependent variable. For exploration of correlations between sNfL levels and continuous variables, we used multiple linear regression with sNfL levels as the dependent variable, and the continuous outcome variable as independent variable. The effect size exp(*B*) is reported for logistic regression analyses. Where relevant, the 95% CI was reported.

## Results

### Patients

The 90 patients were recruited from a clinical trial which showed no difference in clinical outcome between patients treated with 2- or 6-weeks oral doxycycline [12]. We found no significant differences between the two treatment groups regarding age, sex, symptom duration before inclusion, cell count or IgG in CSF, total CCS or sNfL levels, neither at baseline nor at 6-month follow-up (Mann–Whitney *U* test for two independent samples). Therefore, the two treatment groups of the clinical trial were merged for analyses of sNfL levels against clinical parameters.

### Baseline characteristics

The baseline characteristics are shown in Table 1.

**Table 1:** Baseline characteristics, clinical and laboratory findings

	*N* = 90
Sex, *N* female/male	40/50
Age, median years	59 (15)
Symptom duration in days	21 (25)
Definite/possible LNB^a^ *N* (% definite)	75/13 (83)
Cell counts in CSF, millions/liter	110 (167)
Observed tick bite last 6 months Y/N	50/40
Tick bite ever registered Y/N	76/14
Erythema migrans last 6 months Y/N	18/72
sNfL level at baseline, pg/ml	24 (61)

Values are medians (interquartile range), if not stated otherwise

*LNB* Lyme neuroborreliosis, *CSF* cerebrospinal fluid, *sNfL* serum neurofilament light chain, *Y/N* yes/no

^a^Two patients had missing data for antibody index

Out of 90 patients included, 75 patients (83%) had definite LNB according to European Federation of Neurological Societies (EFNS) guidelines. The 13 patients with possible LNB had symptoms typical for LNB, and 12 of them had pleocytosis, but negative Bb antibody index. One patient had positive Bb antibody index without pleocytosis.

### Serum neurofilament light chain

**Fig.1:**
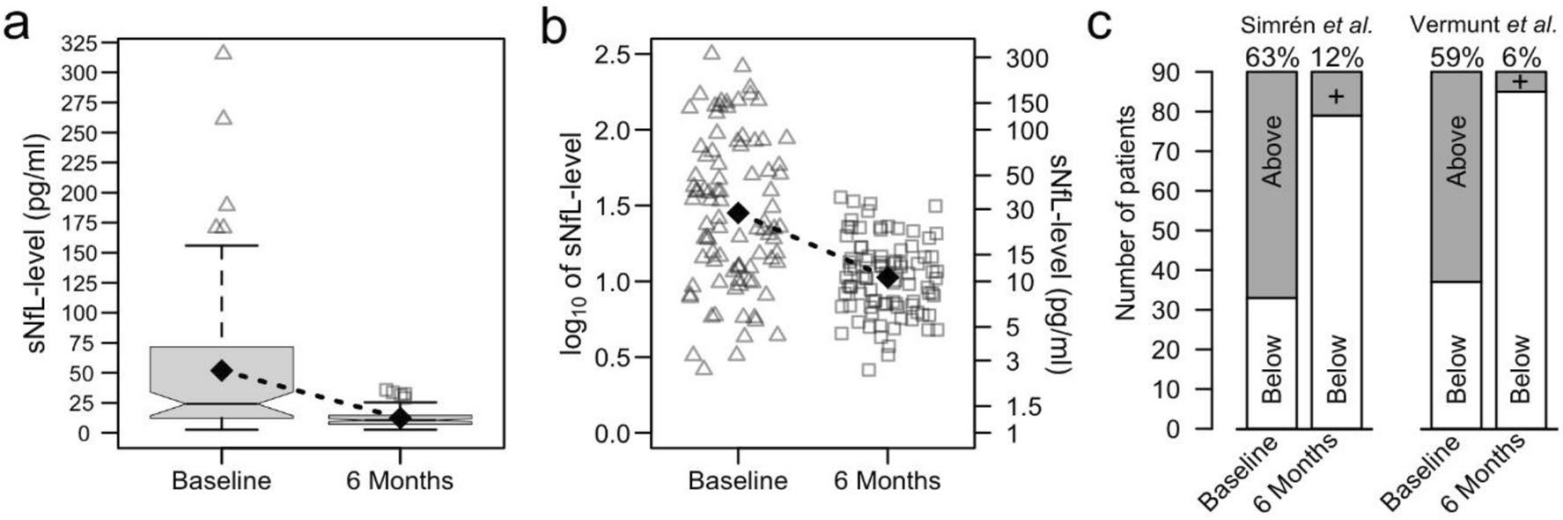
**a** Boxplots of sNfL levels at baseline and 6-month follow-up for the 90 patients in this study. The median sNfL level at baseline (left boxplot) is 24 pg/ml. The interquartile range is 61 pg/ml (box) and mean sNfL level is 52 pg/ml (diamond). For 6-month follow-up (right boxplot), the median sNfL level is 11 pg/ml with interquartile range 8 pg/ml (box), mean sNfL level 1.45 pg/ml (diamond). Whiskers indicate values that exceed 1.5 × IQR from the boxes (outliers). The horizontal lines in the boxplots are medians, notches in the boxes indicate the 95% confidence interval for the median. A dotted line is drawn between the means (black diamonds). **b** Scatterplot of the log_10_-transformed baseline (triangles, left side) and 6-month follow-up (boxes, right side) for the 90 patients in this study. A dotted line is drawn between the means (black diamonds). On the left axis, a log_10_ scale is provided with the sNfL values corresponding to the log_10_-transformed values on the right axis for easier reference. **c** Bargraphs of the number of patients that surpass the normal sNfL threshold according to Simrén et al. [24] (left grouped bars) or patients that are above the 95th percentile of normal values as provided by the calculator of Vermunt et al*.* [25] (right grouped bars). Percentages indicate patients surpassing these respective thresholds

We observed a highly significant and strong reduction in median sNfL levels from 24 pg/ml at baseline, to 11 pg/ml at 6-month follow-up (*p* < 0.001, Wilcoxon signed rank test). The median sNfL reduction was 14 pg/ml (Fig. 1a, b).

There were no significant differences in sNfL levels between the groups with definite or possible LNB (logistic regression, adjusted for age), and no significant correlations between sNfL levels and symptom duration, cell count in CSF or total CCS at the time of inclusion (linear regression). One participant had missing clinical and biochemical information for the follow-up visit, but sNfL levels were available for all patients at both time points.

To evaluate if sNfL levels were elevated in individual patients, we had to consider the fact that sNfL levels increase with age, approximately by 2.2% per year [26]. Although there are no established age-specific cut-offs for normal levels of sNfL in serum, some studies have suggested cut-off limits, and we explored two of these in our material [24, 25]. When using age-adjusted cut-off limits according to Simrén et al. [24], 57 patients (63%) had elevated sNfL at baseline, and 11 patients (12%) had elevated levels at 6 months. The samples that were elevated at 6 months had a median value of 2 pg/ml above the age-specific cut-off limit (95% CI 0.25–6.61 pg/ml). When using the online calculator provided by Vermunt et al. [25], 53 patients (59%) had sNfL levels above the 95th percentile at baseline, and 5 patients (6%) had sNfl levels above the 95th percentile at 6 months (Fig. 1c).

When comparing patients with sNfl levels at or above the 95th percentile with the group below the 95th percentile at baseline, no significant differences were observed regarding duration of symptoms, cell counts in CSF at baseline or CCS total scores at baseline or at follow-up (logistic regression, adjusted for age). However, patients with values at or above the 95th percentile at baseline had significantly higher age and IgG levels in CSF (Table 2). Similarly, when comparing patients with “positive” and “negative” sNfL levels based on the cut-offs suggested by Simrén et al*.,* age and IgG levels in CSF were significantly higher at baseline in patients with a sNfL level above the age-adjusted cut-off. Other analysed parameters did not show significant differences (Table 2, all comparisons by Mann–Whitney *U* test).

**Table 2:** Comparison of patients with elevated sNfL levels and normal sNfL levels, according to two published methods

	Percentiles according to Vermunt et al.			Positive/negative according to Simrén et al.		
	< 95 percentile *N* = 37	≥ 95 percentile *N* = 53	*p* value	Negative *N* = 33	Positive *N* = 57	*p* value
Age, years	53 (49–57)	60 (57–63)	0.011^a^	53 (48–57)	60 (57–63)	0.010^a^
Cell counts in CSF, millions/liter	127 (82–171)	206 (155–258)	0.060	134 (85–183)	196 (147–245)	0.187
CCS baseline	8 (7–10)	10 (9–11)	0.052	8 (7–10)	10 (9–11)	0.059
CCS 6 months	3 (2–4) *N* = 36	3 (2–4)	0.489	3 (2–4) *N* = 32	3 (2–3)	0.791
Symptom duration, days	36 (7–65)	29 (21–37)	0.128	37 (21–37)	29 (5–70)	0.126
IgG in CSF, baseline, mg/L	108 (66–150) *N* = 17	235 (166–305) *N* = 24	0.005^a^	100 (52–147) *N* = 14	225 (162–287) *N* = 27	0.006^a^

All numbers are means (95% CI), unless stated otherwise

Mann–Whitney *U* test applied for statistical testing of difference between groups

*CSF* cerebrospinal fluid, *M/L* millions/liter, *CCS* composite clinical score

^a^Marks significant difference

**Fig. 2:**
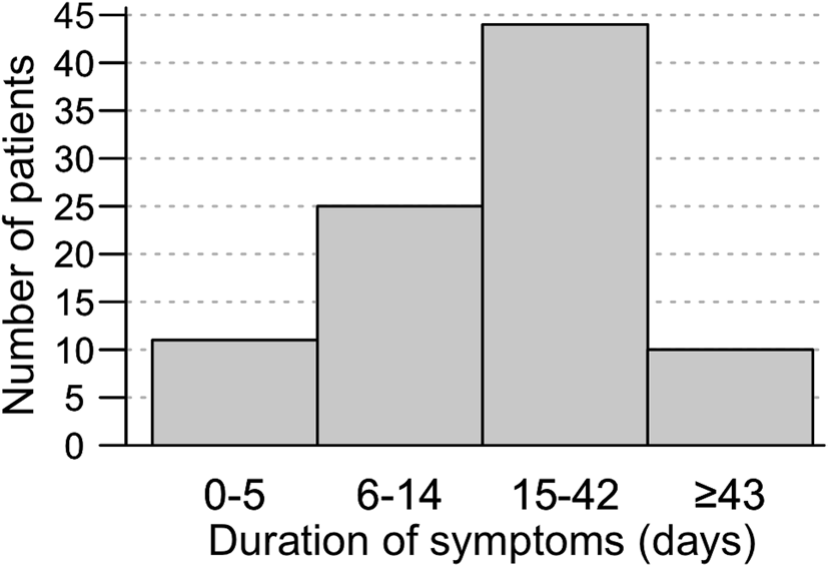
Histogram of number of days with symptoms before inclusion, divided in categories for the 90 patients in the study. Ten patients (11%) had ≥ 43 days of symptoms before inclusion, and eleven patients had < 6 days with symptoms. Median symptom duration was 21 days

When stratifying patients on symptom duration before inclusion and treatment startup (logistic regression adjusted for age, Fig. 2), we observed no association between sNfL levels in the groups with under or more than 42 days treatment delay, which is shown to be associated with persisting symptoms [27, 28].

### Clinical findings

Physician scored sensory and/or motoric findings from cervical, thoracic or lumbar nerve roots were present in 31 patients (34%) at inclusion. The sNfL levels in these patients were significantly higher at baseline compared to the patients without these findings (logistic regression adjusted for age, *p* = 0.033, Table 3). Radicular pain in the abdomen, chest, arms or legs at the time of diagnosis was reported by 67 patients (74%), and 39 of these (43%) had pain as the only radicular symptom. Patients with radicular pain at inclusion did not have different sNfL levels compared to patients without radicular pain (logistic regression adjusted for age, *p* = 0.157 for all patients with pain, *p* = 0.334 for patients with pain only, Table 3).

**Table 3:** Comparison of sNfL levels at baseline in patients with and without listed symptoms in the acute phase

Symptoms and findings *N* with/without	sNfL, median pg/ml (IQR) with finding	sNfL, median pg/ml (IQR) without finding	*p* values, (effect size)
Symptom duration ≥43 days/ < 43 days (10/80)	27 (60)	24 (63)	0.297
Objective radicular findings in spinal nerves (31/59)	50 (133)	23 (35)	0.033^a^ (1.009)
Radicular pain (67/23)	25 (72)	23 (26)	0.157
Radicular pain without objective findings 39/51	20 (46)	34 (73)	0.334
Objective findings in cranial nerves 51/39	24 (38)	34 (78)	0.719
Fatigue and/or malaise 78/12	27 (67)	20 (26)	0.153
Headache 48/42	23 (39)	39 (127)	0.114
CCS > 10 baseline 36/54	51 (82)	20 (27)	0.007^a^ (1.013)

The patient groups are compared with logistic regression, including age as an independent variable (covariate)

*sNfL* serum neurofilament light chain, *CCS* composite clinical score

^a^Marks significant

Cranial nerve affection was registered in 51 patients (57%) at inclusion, with 45 of these having facial palsy. Other cranial nerve symptoms were diplopia, reduced hearing and symptoms registered as “other cranial neuropathies”. The sNfL levels were not significantly different when comparing patients with or without cranial affection, neither at baseline (*p* = 0.719) nor at 6-month follow-up (*p* = 0.123, Table 3). Comparisons were performed by logistic regression, adjusted for age.

### Subjective symptoms not related to an organ system

In total, 83 out of 90 patients reported fatigue, malaise, headache or problems with memory or concentration at the time of inclusion. Fatigue and/or malaise was reported by 78 patients, headache by 48 patients, and memory/concentration problems by 29 patients. The sNfL levels did not differ between patients with or without these symptoms, neither at baseline nor at 6-month follow-up (logistic regression adjusted for age, Table 3).

### Composite clinical score

The CCS at inclusion varied from 3 to 27 points, with a median of 9 points. The 36 patients with a CCS > 10 points had significantly higher sNfL levels compared to patients with CCS ≤ 10 (logistic regression adjusted for age, *p* = 0.007, Table 3). At 6-month follow-up, only one patient had CCS > 10 points.

**Fig. 3:**
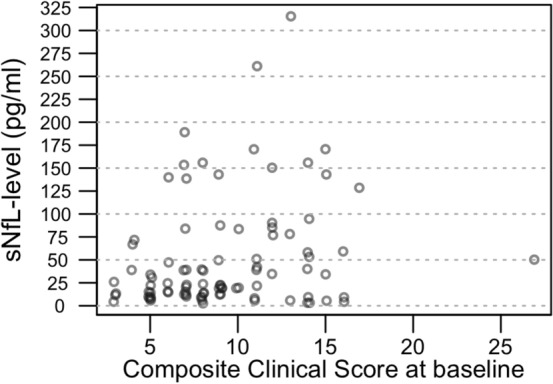
Scatterplot of sNfL levels at baseline plotted against the composite clinical score

At 6-month follow-up, 18 patients had symptoms influencing their daily life. There were no significant differences in sNfL levels in this patient group compared to patients with only mild scores, neither at baseline (*p* = 0.972) nor at 6 months (*p* = 0.278) (Table 3 and Fig. 3, comparisons made by logistic regression adjusted for age).

## Discussion

Our results from a large, prospective well-defined cohort of patients with LNB show that sNfL is a potential biomarker for peripheral nerve affection.

We found that sNfL levels normalized for nearly all patients 6 months after diagnosis and treatment. The prognostic significance of sNfL levels at baseline with respect to treatment outcome at 6 months was insignificant, confirming the findings from an earlier study including 36 LNB patients [21]. We observed that patients with CCS > 10 at baseline had significantly higher sNfL levels than patients with CCS ≤ 10. This may be explained by different degree of nervous tissue affection in the two CCS categories. Since we do not have a direct measure of the volume of affected nerve tissue, this hypothesis needs to be explored in a different study design in which, e.g., imaging modalities are used to quantify the volume of affected nerve tissue. We also found that IgG levels in CSF were higher in patients with elevated sNfL levels, adjusted for age. IgG is not normally present at measurable levels in the CSF, so an elevated level could be a sign of intrathecal inflammation.

One interesting and highly significant finding was that physician scored motor and/or sensory radicular findings from the spinal nerve roots were associated with higher sNfL levels at baseline as compared to patients without these findings. Pain as the only radicular symptom did not show such an association. The biological mechanism underlying this phenomenon is unclear, but one explanation could be that the nerve damage is more widespread with paresis and/or objective sensory findings than with pain alone.

We note that not all patients had elevated sNfL at inclusion. Patients with sNfL ≤ 10 pg/ml at baseline were significantly younger as compared to patients with levels > 10 pg/ml. There is a need for more insights in how age affects the observed sNfL levels, illustrated by several initiatives to establish age-related trends of sNfL and normal values for different age groups [24, 25, 29–31]. In our study, we did not have an age-matched control group, so we cannot exclude age as a factor in the observed lower levels, or whether this is caused by absence of nerve inflammation or nerve damage.

Since we do not have quantitative data on the level of nerve inflammation, we cannot conclude that sNfL is a biomarker for ongoing inflammation in neurons. We did not find significant association between sNfL levels and symptom duration, suggesting that sNfL is an LNB biomarker not only in the early phase of the disease, but also when there is an ongoing and untreated inflammation over weeks. To verify that sNfL is a biomarker of LNB-related inflammation of the nervous system, a model system that assesses longitudinal sNfL level measurements over the course of untreated LNB is warranted.

Since even patients with very high sNfL levels (> 100 pg/ml) returned to values within normal range after treatment for LNB, it can be hypothesized that the nerve damage is limited to the acute and untreated phase of the disease. We conclude that sNfL is not suitable for predicting patient outcomes 6 months after completed treatment.

Patients with residual LNB-attributed symptoms and findings did not exhibit elevated sNfL levels 6 months after diagnosis and treatment. Given that all participants in our study underwent treatment for LNB, this observation suggests that lingering symptoms are not directly associated with ongoing processes involving neuronal damage that leads to release of NfL. In the absence of sNfL indicative of ongoing neuronal damage, it can be hypothesized that these symptoms are a sign of sequelae rather than ongoing inflammation and nerve damage.

In contrast to the findings in spinal nerve roots, patients with affection of cranial nerves attributed to LNB did not have higher sNfL levels than patients without cranial nerve involvement. This is interesting in the light of a recent MRI-study of patients with LNB, performed by Lindland et al*.* [32]. They found that 51% (19/37) of the patients with MRI signs of facial nerve inflammation and 94% (16/17) of the patients with MRI signs of oculomotor/abducens nerve inflammation did not have clinical signs of corresponding cranial nerve palsies. They conclude that subclinical cranial neuritis is to be expected in a substantial share of patients [32]. Eventually, the presence of subclinical cranial neuritis without corresponding symptoms can explain why we did not observe associations between sNfL levels and affection of cranial nerves.

Neurofilament light levels in serum reflect leakage of NfL from the CNS into the blood circulation. Therefore, the measured sNfL is only a proxy marker for the NfL levels in the nervous compartment. Previous studies have shown a high level of correlation between the NfL levels in serum and CSF, indicating that sNfL can be used to monitor the NfL levels in the nervous compartment [33]. However, we acknowledge that myelinated axons are rich in neurofilament light chains, so the NfL levels measured in serum can also be derived from the PNS. There is need for better understanding the pathophysiology and the distribution of nervous tissue inflammation in LNB.

## Conclusion

To our knowledge, this is the first study that identifies a possible relation between sNfL levels and spinal radiculopathy in the acute phase of LNB.

Our study results do not support the use of sNfL as a prognostic marker in LNB. However, sNfL seems to be associated with the level of inflammation of peripheral nerves in the acute phase of LNB.

## Data Availability

The data that support the findings of this study are not openly available due to privacy or ethical restrictions. Data are available from the corresponding author upon reasonable request, after approval of a proposal to the BorrSci study group.
